# Resolving the taxonomic enigma of the iconic game fish, the hump-backed mahseer from the Western Ghats biodiversity hotspot, India

**DOI:** 10.1371/journal.pone.0199328

**Published:** 2018-06-20

**Authors:** Adrian C. Pinder, Arunachalam Manimekalan, J. D. Marcus Knight, Prasannan Krishnankutty, J. Robert Britton, Siby Philip, Neelesh Dahanukar, Rajeev Raghavan

**Affiliations:** 1 Faculty of Science and Technology, Bournemouth University, Dorset, United Kingdom; 2 Mahseer Trust, Freshwater Biological Association, Wareham, Dorset, United Kingdom; 3 Department of Environmental Sciences, Bharathiar University, Coimbatore, India; 4 India Ministry of Environment Forest and Climate Change, Government of India, New Delhi, India; 5 Department of Zoology, Mahatma Gandhi College, Thiruvananthapuram, India; 6 Department of Zoology, Nirmalagiri College, Kannur, India; 7 Indian Institute of Science Education and Research, Pune, India; 8 Zoo Outreach Organization (ZOO), Coimbatore, India; 9 Department of Fisheries Resource Management, Kerala University of Fisheries and Ocean Studies Kochi, Kerala, India; University of Delhi, INDIA

## Abstract

Growing to lengths and weights exceeding 1.5 m and 45 kg, the hump-backed mahseer fish of the Western Ghats biodiversity hotspot, India, is an iconic, mega-faunal species that is globally recognized as a premier freshwater game fish. Despite reports of their high extinction risk, conservation approaches are currently constrained by their lack of valid taxonomic identity. Using an integrative approach, incorporating morphology, molecular analysis and historical photographs, this fish can now be revealed to be conspecific with *Tor remadevii*, a species lacking a common name, that was initially, but poorly, described in 2007 from the River Pambar, a tributary of the River Cauvery in Kerala. Currently known to be endemic and restricted to the River Cauvery basin in the Western Ghats, *T*. *remadevii* is distinguished from congeners by its prominent hump originating above the pre-opercle and extending to the origin of the dorsal fin, a well-developed mandible resulting in a terminal or slightly superior mouth position, and the dorsal orientation of the eyes. While body colouration varies (silver, bronze, greenish) and is not considered a reliable diagnostic character, orange coloration of the caudal fin (sometimes extending to all fins) is considered a consistent characteristic. Having been first brought to the attention of the scientific community in 1849, and the recreational angling (game fishing) community in 1873, it has taken over 150 years to finally provide this iconic fish with a valid scientific name. This taxonomic clarity should now assist development and delivery of urgent conservation actions commensurate with their extinction risk.

## Introduction

Freshwater megafauna (defined as species with adult body weights of at least 30 kg) occur in large rivers and lakes of every continent except Antarctica [1]. These megafauna comprise one of the world’s most vulnerable groups of vertebrates to extinction, with 58% of species at threat from stressors including overexploitation, habitat alteration and pollution [1–2]. Despite this, for many freshwater mega-fauna, knowledge on their taxonomy, natural history and threats remain incomplete, as despite their body sizes providing high anthropogenic interest, some species have only recently been described [3], while the identity of others remain to be elucidated [4].

**Fig 1:**
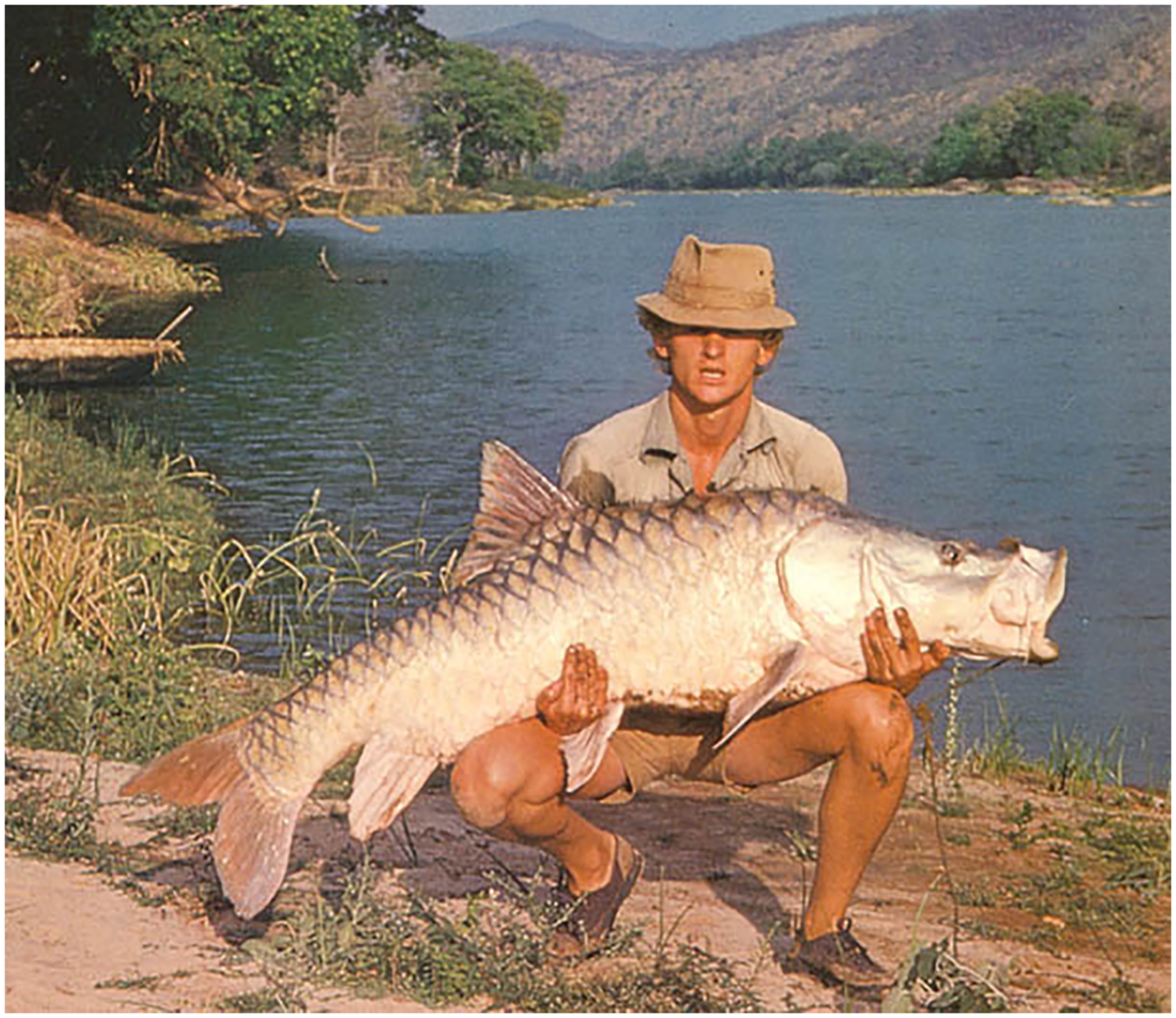
Adult Cauvery hump-backed mahseer, *Tor remadevii* captured by Martin Clark, 1978 [Photo Credit: Trans World Fishing Team].

With validated body weights exceeding 45 kg [5], the hump-backed mahseer of the River Cauvery (Western Ghats, India) represents the largest of all known mahseers of the *Tor* genus (Fig 1). Globally recognized by recreational fishers as an iconic game fish for over a century [6], it was initially brought to their attention in 1873, under the nom de plume ‘Barbus tor’ [6], with documentation of a world record specimen of 119 lbs (54 kg) captured in 1921 from the River Kabini, a tributary of the River Cauvery [7]. Following Indian independence in 1947, the fish was largely forgotten until a resurgence in recreational angling interest and subsequent development of catch-and-release fisheries in the main River Cauvery in the early 1970s [8–9]. These fisheries subsequently became world famous for the size of mahseer they produced [8–9] and were also recognized for the socio-economic benefits afforded to poor rural communities via ecotourism based employment opportunities [8].

Despite this long-term interest in the species, the hump-backed mahseer continued to be erroneously known under the names *Barbus mussullah* and *Tor mussullah*, both in scientific [10–13] as well as in popular literature [14]. This continued until Knight and coworkers [15–16] stabilized the use of the name ‘mussullah’ to a species of the cyprinid genus *Hypselobarbus*. However, this taxonomic revision continued to leave the hump-backed mahseer without a valid scientific identity, thus denying the formal recognition required to undertake IUCN Red List assessment and afford protection commensurate with their apparent high extinction risk [5].

A new species of mahseer, *Tor remadevii* was described in 2007 from the River Pambar, the southern-most tributary of the River Cauvery [17]. This was based on the examination of 19 juvenile specimens (lengths 113.64mm to 331.82mm) [17]. However, neither a photograph of a live/preserved specimen, nor an illustration, accompanied the description, with no comparison to material from congeners. The description thus relied entirely on morphological measurements and counts available in the literature [17]. Despite these issues and the limited sample size, many of the characters were consistent with those observed from images of the hump-backed mahseer caught by recreational fishers in the River Cauvery (e.g. body shape: “dorsal profile has a moderate to prominent hump between the head region and the dorsal fin”), colouration: (“fins reddish with black patches”; “younger specimens with red orange fins”) and a “distinctively longer mandible than other Southern Indian *Tor* species, resulting in a terminal/posterior and slightly upturned mouth”). Consequently, given the outstanding requirement to resolve the taxonomic identity and assist the conservation of the hump-backed mahseer, the aim of this study was to 1) apply morphological and molecular analyses to test whether the hump-backed mahseer is distinct from the currently known South Indian *Tor* species, and whether it is conspecific with *T*. *remadevii*, 2) provide definitive morphological characters which can be reliably used to identify this species from congeners in the field, and 3) provide notes on current knowledge relating to distribution and habitat utilization.

## Materials and methods

### Ethics statement

Samples for the present study originated from three sources: (1) tissue samples (as fin-clips) for molecular analyses obtained from cast-net sampling and catch-and-release angling, where the specimens were released back in the wild, (2) voucher specimens collected from inland fish markets (from where dead specimens were purchased), and (3) voucher specimens collected from stream habitats inside protected areas. Permissions for collecting specimens inside protected areas were issued by the Department of Forests and Wildlife, Government of Kerala to Rajeev Raghavan (WL12-8550/2009) and Government of Tamil Nadu (WL5 (A) /26789/2017) to A. Manimekalan. Immediately upon capture using a cast net or rod-and-line, specimens were euthanized (anesthetic overdose; tricaine methanesulfonate, MS222; following the guidelines developed by the American Society of Ichthyologists and Herpetologists (ASIH) (http://www.asih.org/pubs/; issued 2013)). Samples of pelvic fin tissue were taken and stored in absolute ethanol. Voucher specimens were preserved whole in either 5% formalin or 70% ethanol. Institutional ethics committee of Mahseer Trust approved the design and implementation of the study (MTE/ 17/01). In-country (India) ethical approvals were not required as no experimentation or manipulations were carried out. All molecular genetic work was completed within India and no specimens or fish tissues were taken out of the country. Voucher specimens were primarily deposited in national and/or regional repositories. Individual participants who appear in the Figures in this manuscript have given written informed consent (as outlined in PLOS consent form) to publish these case details.

### Specimen collection and vouchers

**Fig 2:**
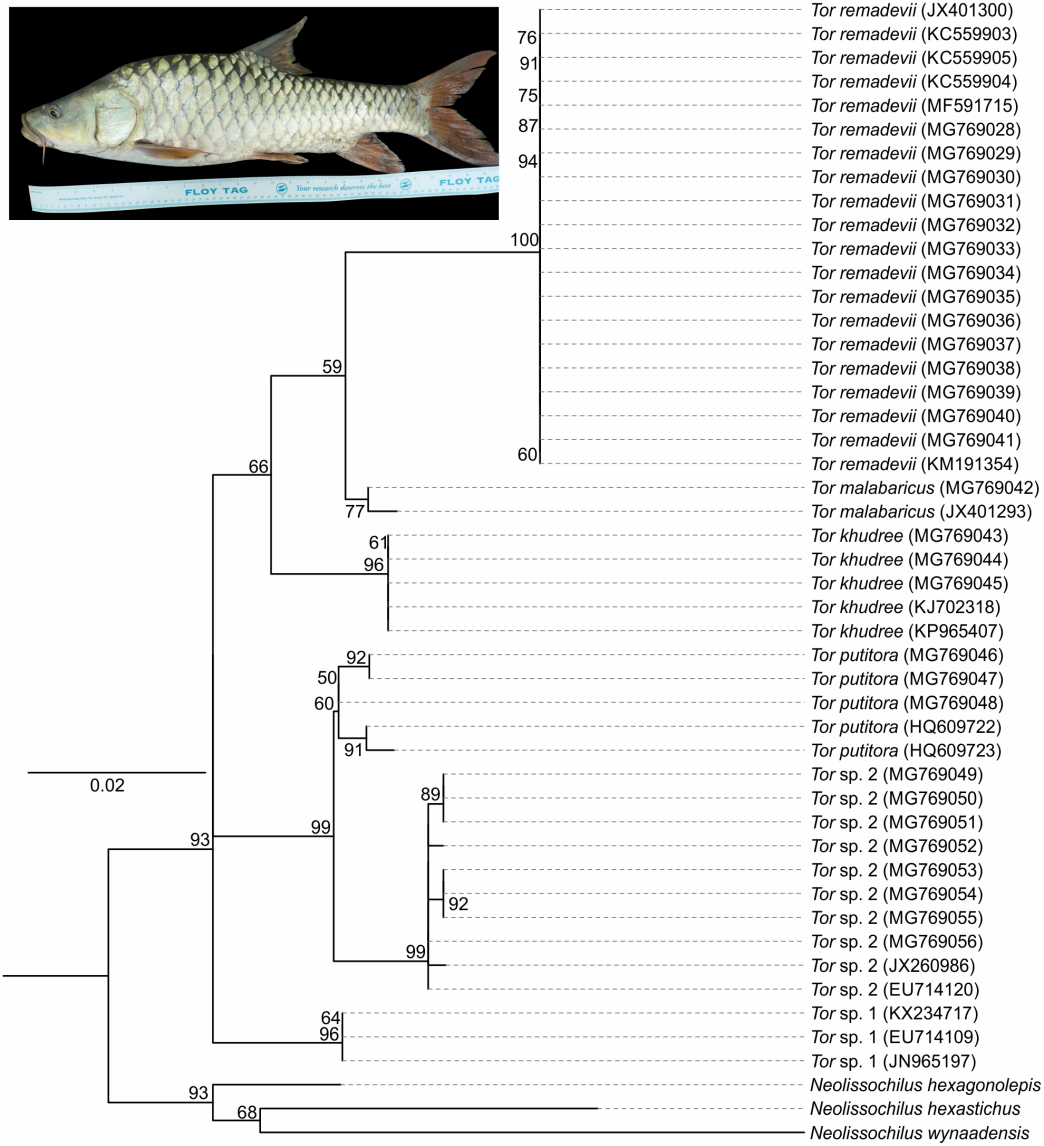
Maximum likelihood phylogenetic tree based on cox1 sequences of mahseer species occurring in India (*Tor* sp 1 represent individuals not matching any of the described species from India and could potentially comprise new species, *Tor* sp. 2 are sequences available in GenBank with uncertain identities, i.e. under different species names). Species of *Neolissochilius* are used as outgroup. Values along the nodes are percentage bootstraps for 1000 iterations.

Topotypic specimens of mahseer species were collected from various rivers in India: *Tor khudree* from River Krishna and its tributaries in Maharashtra, *Tor malabaricus* from River Chaliyar in Kerala, *T*. *remadevii* from River Pambar in Kerala, and the hump-backed mahseer from River Moyar in Tamil Nadu. The fishes were preserved in 10% formaldehyde and transferred to 5% formaldehyde or 70% ethanol for long-term storage. Fin clips from topotypic *Tor putitora* from River Teesta in West Bengal, and hump-backed mahseer from the River Cauvery at Dubare, Karnataka and River Moyar in Tamil Nadu were taken. In addition, fin clips from a yet-to-be identified mahseer species from River Vaitarna, Harkul Reservoir, Krishna River in Maharashtra and Forbes Sagar Lake in Karnataka (see *Tor* sp 1 in Fig 2) were also collected following their sampling by catch-and-release angling. Tissue samples were preserved in absolute ethanol. Voucher specimens are in the museum collections of the Zoological Survey of India, Kolkata (ZSI); Zoological Survey of India—Southern Regional Center, Chennai, India (ZSI-SRC); Zoological Survey of India—Western Regional Center, Pune, India (ZSI-WGRS); Kerala University of Fisheries and Ocean Studies, Kochi, India (KUFOS); Department of Aquatic Biology and Fisheries, University of Kerala, Thiruvananthapuram, Kerala (DABFUK); and in the private collections of J.D. Marcus Knight (MKC).

### Comparative material examined for morphometric analysis

*Tor malabaricus*: 5 ex, MKC 450, 196.6–231.7mm SL, Ivarnadu, Payaswini River, Karnataka, India (12.522°N & 75.425°E); collected by A Rai, August 2014.

*Tor kulkarnii*: Holotype, ZSI F2710, 220.0mm SL, Nashik, Darna River, between Sawnuri and Beladgaon, Deolali, Maharashtra, India (19.929°N & 73.856°E); collected by AGL Fraser, 29 April 1936; paratypes, ZSI F2711, 3 ex., 103.2–197.0mm SL, same data as holotype.

*Tor khudree*: ZSI-WRC P/2451, 1 ex, 121.9mm SL, Neera River, Bhor, Pune, Maharashtra, India (18.152°N & 73.829°E); collected by N Dahanukar and M Paingankar, 20 August 2010; ZSI-WRC P/3067, 6 ex. 106.1–171.2mm SL, Krishna River, Wai, Satara, Maharashtra, India (17.991°N & 73.786°E); collected by N Dahanukar and M Paingankar, 2 February 2011; ZSI-WRC P/3072, 5 ex. 77.4–151.2mm SL, Krishna River, Wai, Satara, Maharashtra, India (17.991°N & 73.786°E); collected by N Dahanukar and M Paingankar, 18 February 2011; ZSI-WRC P/3071, 7 ex. 51.5–66.7mm SL, Koyna River, Patan, Satara, Maharashtra, India (17.367°N & 73.903°E); collected by N Dahanukar and M Paingankar, 1 July 2007.

### Morphometric analysis

Point to point measurements were made using digital calipers, to the nearest 0.1 mm, based on standard methods employed for cyprinid fishes [18] and *Tor* mahseer [19]. Morphometric data used in the study is available online on figshare (https://doi.org/10.6084/m9.figshare.6085982). Statistical analysis of the morphometric data was performed on size-adjusted measurements of subunits of the body expressed as proportions of standard length and subunits of head expressed as proportions of head length. The null hypothesis that the data were multivariate-normal was checked [20]. Multivariate Analysis of Variance (MANOVA) was performed to test whether the populations of different species (see comparative material examined) formed significantly different clusters [21] using Pillay’s trace statistic [22]. Mahalanobis distances [22] between pairs of individuals were calculated and used for computing Fisher’s distances (distance between the centroids of the clusters, divided by the sum of their standard deviations) between two clusters to check if the species clusters were significantly different from each other. Statistical analyses were performed in PAST 3.16 [23].

### Molecular analysis

DNA extraction, PCR amplification for cytochrome oxidase subunit 1 (cox1) gene and sequencing protocols were as per [24]. Sequences were checked using BLAST [25] and the sequences generated as part of this work deposited in GenBank under the accession numbers MG769028 to MG769056 (S1 Table). *Neolissochilus* species were used as outgroup based on earlier study [26]. Gene sequences were aligned using MUSCLE [27], and raw (p) distances for cox1 between pairs of sequences were calculated in MEGA 7 [28]. The best-fit partition model and the substitution model was found using the IQTree software [29] based on the Bayesian Information Criterion (BIC) [30–31]. Maximum likelihood analysis based on best partition scheme was performed in IQ-Tree [28] with ultrafast bootstrap support for 1000 iterations [32]. The phylogenetic tree was edited in FigTree v1.4.2 [33].

## Results

### Molecular analysis

The results suggested that the best partition scheme was Tamura & Nei’s [34] model with invariant sites (TN+I, BIC = 3622.967, lnL = -1580.211, df = 71) for combined partition of all three codon positions. Topotypic *T*. *remadevii* formed a monophyletic clade with the hump-backed mahseer collected from widely distributed populations from within the Cauvery River system (Fig 2; Table 1). Genetic distance between *T*. *remadevii* and other species of *Tor* from peninsular India ranged between 2.3 and 4.6% (Table 1).

**Table 1:** Pairwise percentage raw (p) genetic distances between *Tor* species.

	[1]	[2]	[3]	[4]	[5]	[6]
*Tor remadeviii* [1]	0.0–0.0					
*Tor malabaricus* [2]	2.3–2.8	0.3–0.3				
*Tor khudree* [3]	2.7–3.2	1.6–2.0	0.0–0.0			
*Tor putitora* [4]	2.7–4.3	2.0–3.5	2.2–3.0	0.0–1.0		
*Tor* sp2 [5]	3.3–4.6	2.1–3.4	3.1–3.8	1.1–2.2	0.0–0.4	
*Tor* sp1 [6]	2.8–3.6	1.8–3.0	2.8–3.3	2.4–2.9	2.8–3.4	0.0–0.0

### Morphometrics

**Fig 3:**
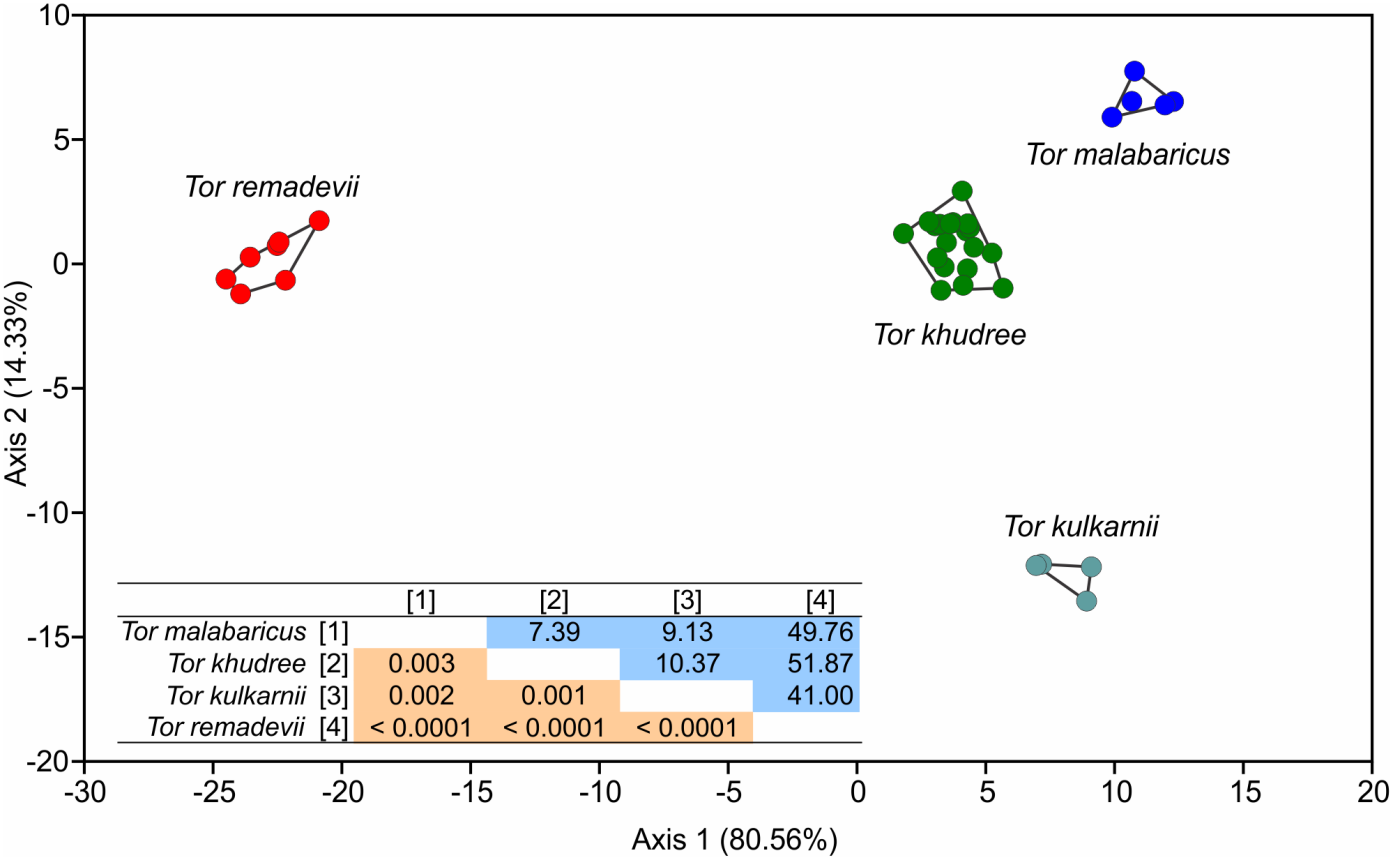
Discriminant analysis of the four peninsular Indian *Tor* species. Fisher's distances between clusters (blue cells) and associated p values (red cells) are provided in inset. Values in parenthesis are the percentage variation explained by each discriminant axis.

Morphometric data were multivariate normal (Doornik and Hansen omnibus, Ep = 55.11, P = 0.168). The four peninsular Indian species of *Tor* formed distinct clusters (Fig 3), with *T*. *remadevii* distinguished based on comparatively larger pre-anal length, head length, pre-ventral length, pre-pectoral length and pre-dorsal length, and comparatively smaller dorsal to caudal length, head length and inter-orbital length (Table 2). The specimens that make up the *T*. *remadevii* group/clade includes the type material of the species (ZSI-WGRS V/F 13119a and 13119b) as well as freshly collected specimens from the River Moyar (see section on comparative material below; Table 3) (ZSI-SRS F 9145, 9148, 9149, 9150).

**Table 2:** Factor loading on the first two axes of discriminant analysis.

Character	Axis 1	Axis 2
Head length	-0.19	0.08
Snout length	0.08	-0.12
Inter orbital length	0.32	0.11
Eye diameter	0.18	0.06
Head depth	0.09	-0.22
Head width	0.41	-0.40
Pre-dorsal length	-0.11	-0.02
Dorsal to caudal distance	0.64	0.07
Pre-pectoral length	-0.16	0.01
Pre-ventral length	-0.18	0.00
Pre-anal length	-0.22	0.05
Caudal-peduncle length	-0.03	-0.07
Caudal-peduncle depth	0.03	0.01
Dorsal-fin length	-0.07	-0.01
Dorsal-fin base	0.01	-0.02
Pectoral-fin length	-0.01	0.16
Ventral-fin length	-0.01	0.13
Anal-fin length	-0.02	0.21
Anal-fin base	-0.01	0.06
Body depth (D)	0.05	-0.08
Body depth (A)	0.06	-0.03
Body width (D)	-0.01	0.14
Body width (A)	0.01	0.04

**Table 3:** Morphometric data of *Tor remadevii* type and comparative material.

Characters	Holotype	Paratypes		Comparative material (ZSI-SRS)			
		#1	#2	F9148	F9149	F9150	F9145
Standard length (SL, mm)	217.1	194.1	168.0	356.0	369.0	487.0	572.0
Head length (HL, mm)	66.0	63.0	60.5	112.8	117.2	159.0	182.4
%SL							
Head length	30.4	32.5	36.0	31.7	31.8	32.6	31.9
Pre-dorsal length	54.4	52.1	57.1	56.2	51.5	55.0	54.9
Dorsal to caudal distance	30.4	33.0	33.3	33.7	36.3	36.3	32.3
Pre-pectoral length	29.0	31.4	34.0	30.9	29.6	30.3	30.2
Pre-ventral length	53.5	56.8	58.3	58.4	58.3	57.7	56.5
Pre-anal length	82.5	88.8	82.2	84.3	84.6	84.2	81.3
Caudal-peduncle length	19.8	24.2	24.1	17.9	16.7	18.3	15.4
Caudal-peduncle depth	12.0	12.4	13.1	10.8	9.1	10.4	9.9
Dorsal-fin length	27.2	29.4	30.4	23.6	23.3	21.1	21.0
Dorsal-fin base	14.7	15.0	14.3	12.5	12.6	11.3	12.6
Pectoral-fin length	21.2	21.1	20.3	18.5	19.3	19.5	20.1
Ventral-fin length	18.9	18.6	19.1	17.0	17.2	17.2	16.6
Anal-fin length	20.8	20.7	19.7	16.0	18.3	17.6	18.2
Anal-fin base	5.6	7.3	7.2	7.7	7.2	7.2	7.1
Body depth (D)	26.7	28.9	31.6	25.9	26.5	24.5	24.8
Body depth (A)	17.1	19.1	19.1	17.4	16.1	15.9	15.8
Body width (D)	14.0	14.4	13.7	14.6	14.2	15.1	16.2
Body width (A)	9.7	8.8	8.4	8.6	8.3	9.6	11.7
% HL							
Snout length	30.4	32.7	31.5	32.0	29.0	30.6	29.3
Inter-orbital length	28.9	20.7	28.2	24.0	22.6	21.7	23.5
Eye diameter	21.3	19.1	19.9	14.1	14.5	12.2	11.9
Head depth	57.6	50.8	52.9	71.4	76.1	69.9	75.6
Head width	41.0	36.5	33.7	43.0	41.6	46.3	48.2

### Taxonomy

*Tor remadevii* Kurup & Radhakrishnan 2007

(Figs 1 and 4–6)

**Fig 4:**
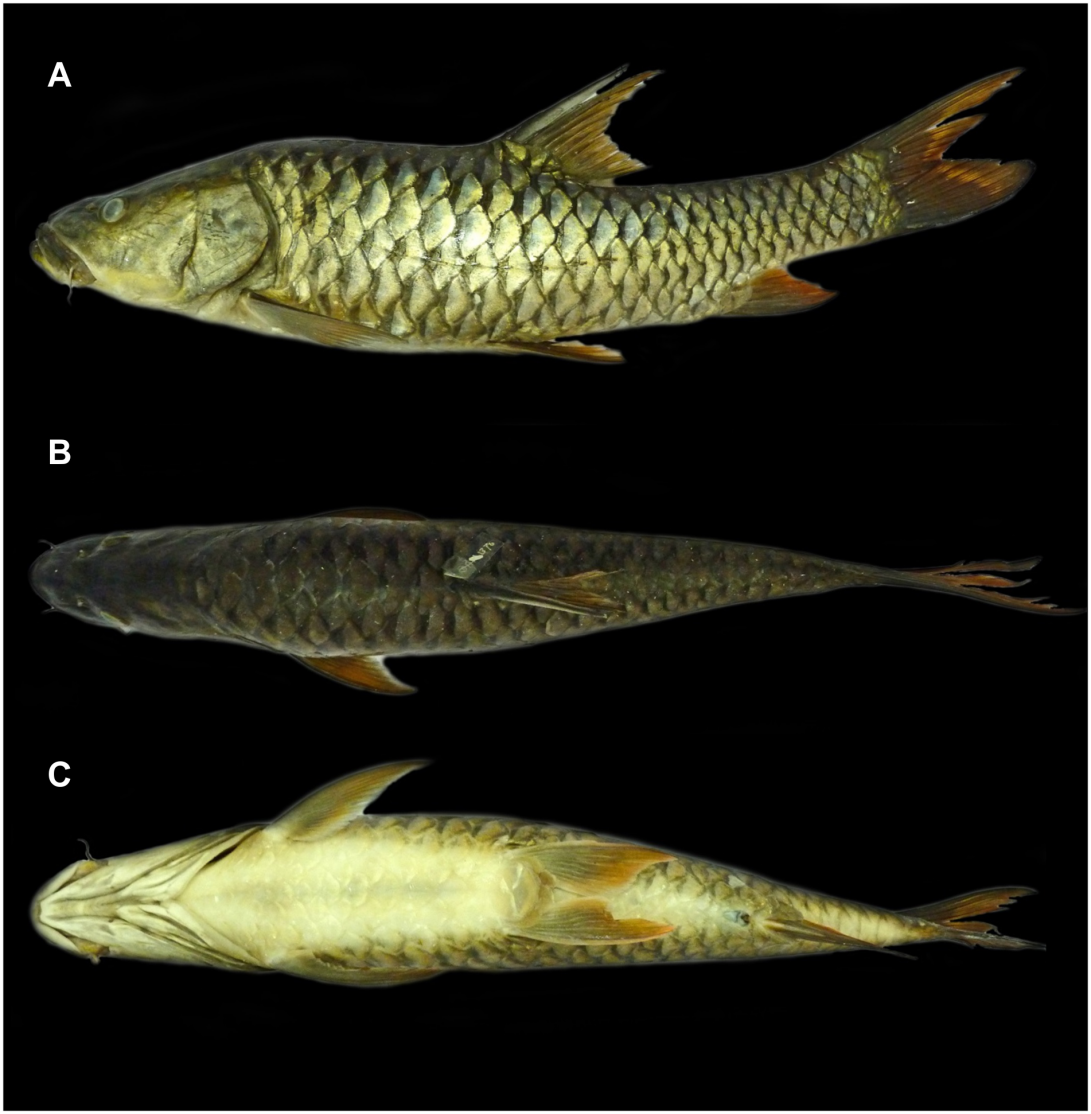
Lateral (A), dorsal (B) and ventral (C) view of *Tor remadevii* (ZSI F-9150, 487 mm SL) collected from the River Moyar, India.

**Fig 5:**
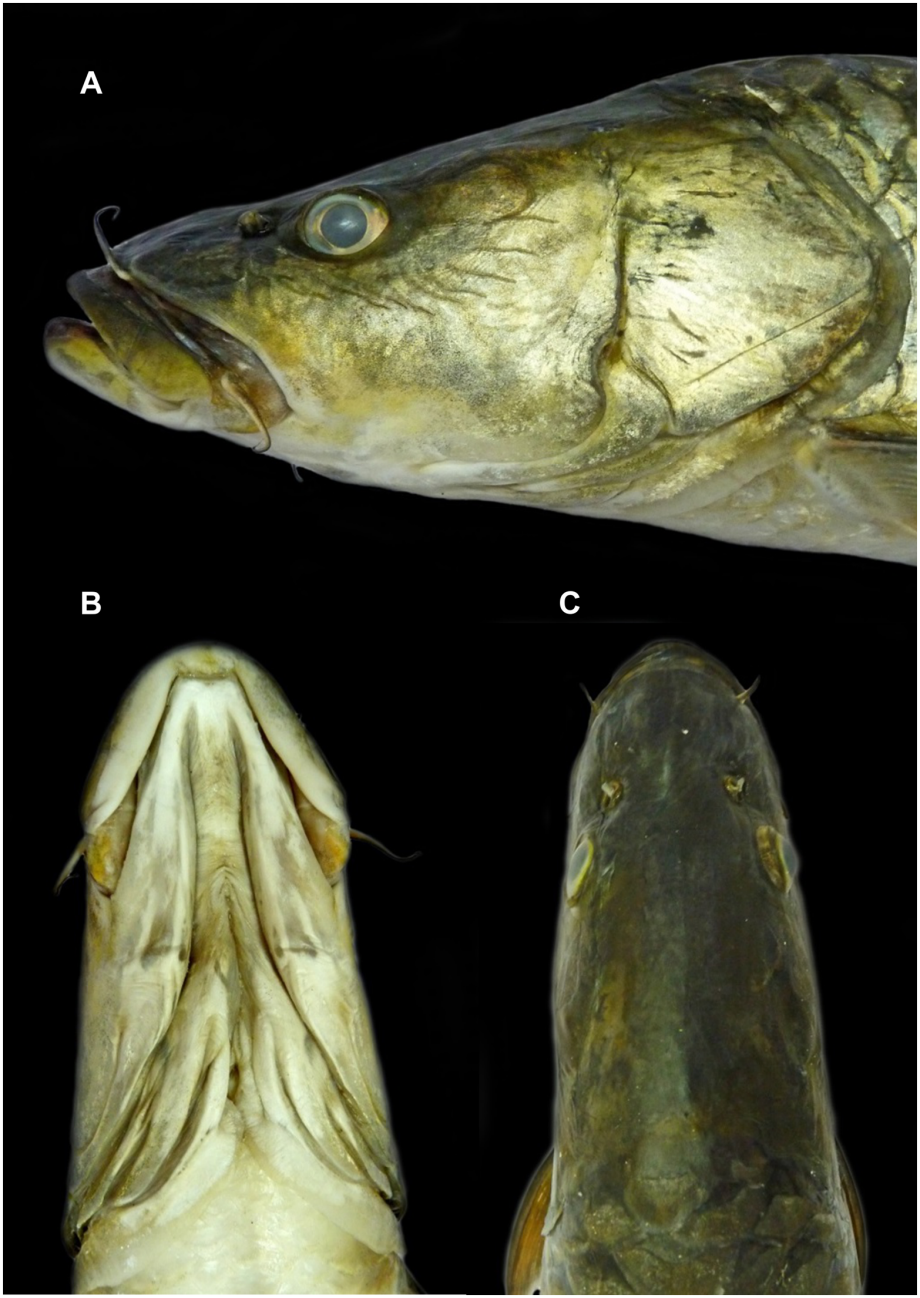
Lateral (A), ventral (B) and dorsal (C) view of the head region of *Tor remadevii* (ZSI F-9150, 487 mm SL) collected from the River Moyar, India.

**Fig 6:**
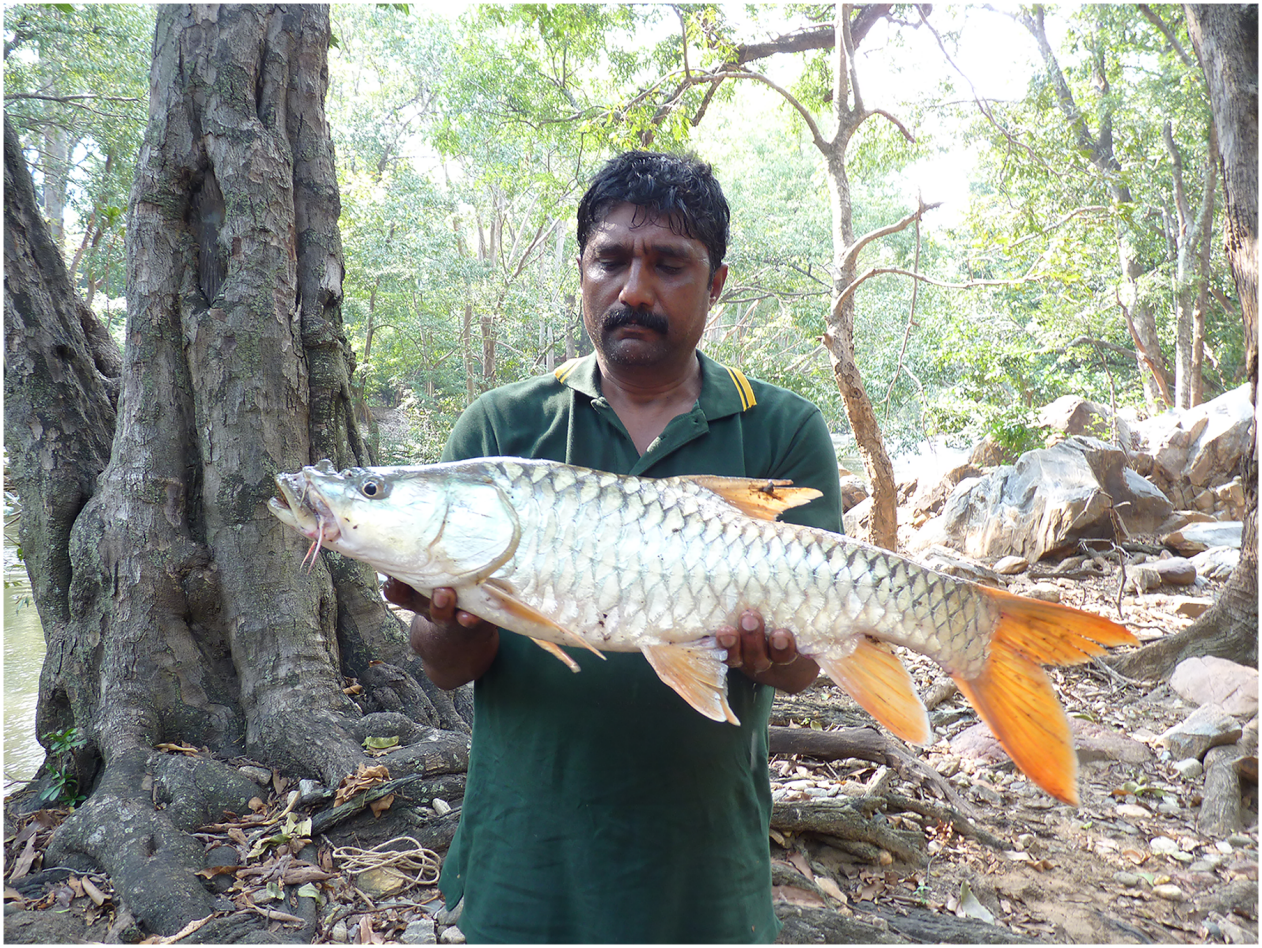
Freshly caught adult *Tor remadevii* from the River Moyar, India, showing the characteristic orange coloured fins.

### Material examined

Type material: ZSI-WGRS V/F 13119a (holotype) and 13119b (paratypes), 3 ex, 168.00–217.063mm SL, River Pambar, Champakkad, Kerala, India; collected by KV Radhakrishnan, 18 May 2004.

Additional material: ZSI-SRS F 9145, 9148, 9149, 9150, 4ex, 356–487mm SL, River Moyar, Thengumarahada, Tamil Nadu, India (11.614°N & 76.740°E; 474m ASL); collected by A Manimekalan, 6–7 October 2017; KUFOS-PK-2016.100.1, 1ex, 84mm SL, Pambar River, Chinnar Check Post, Chinnar Wildlife Sanctuary, Kerala, India (10.353°N, 77.216°E, 454m ASL); collected by P. Krishnankutty, 12 October 2016.

### Diagnosis

*Tor remadevii* can be distinguished from all its congeners by the following combination of characters: large adult body size (≥1500mm Total Length/TL and 45kg), dorsal orientation of eyes not visible from ventral aspect, shorter inter-orbital distance (7.1–9.6% of Standard Length/SL), a distinctive kink in the profile of the pre-opercle and a well-developed mandible extending to either equal distance or anterior of the maxilla, resulting in a terminal or slightly superior mouth position (Fig 5).

### Description

A large sized *Tor* attaining a maximum size of 1500mm TL. For general shape and appearance see Figs 1, 2 and 4–6. Morphometric data are provided in Table 3.

Consistent with the common name, the dorsal profile of *T*. *remadevii* exhibits a prominent hump originating above the pre-opercle and extending to the origin of the dorsal fin. Dorsal fin with 4 unbranched and 9 branched rays, the fourth unbranched ray forming a strong smooth spine. Dorsal-fin origin directly above the pelvic-fin origin. Pelvic fin with one un-branched and 7–8 branched rays. Anal fin with two un-branched and five branched rays. Pectoral fin with one un-branched and 14–15 branched rays. Lateral line complete, with 24–29 scales. Transverse scales from dorsal-fin origin to ventral-fin origin ½3/1/2½. Pre-dorsal scales 7–8. In contrast with the description [17], dorsal-fin height less than and not exceeding 91% of dorsal body-depth. Consistent with other species of *Tor*, pharyngeal teeth display a 5,3,2:2,3,5 ratio.

#### Colouration

Live specimens of *T*. *remadevii* from the River Moyar display contrasting dorsal and lateral body colouration, from deep bronze to metallic greens. Bright orange fins (Fig 6) were consistent in all specimens examined. Photographic records captured by anglers from the main stem of the River Cauvery exhibit body colouration ranging from silver to deep bronze, with orange colouration of fins always evident in caudal fin as a minimum. Colour of the remaining fins range between deep orange and bluish grey. With the exception of fin-colour, observed variations suggest that body colouration may not be a reliable diagnostic character.

### Distribution

**Fig 7:**
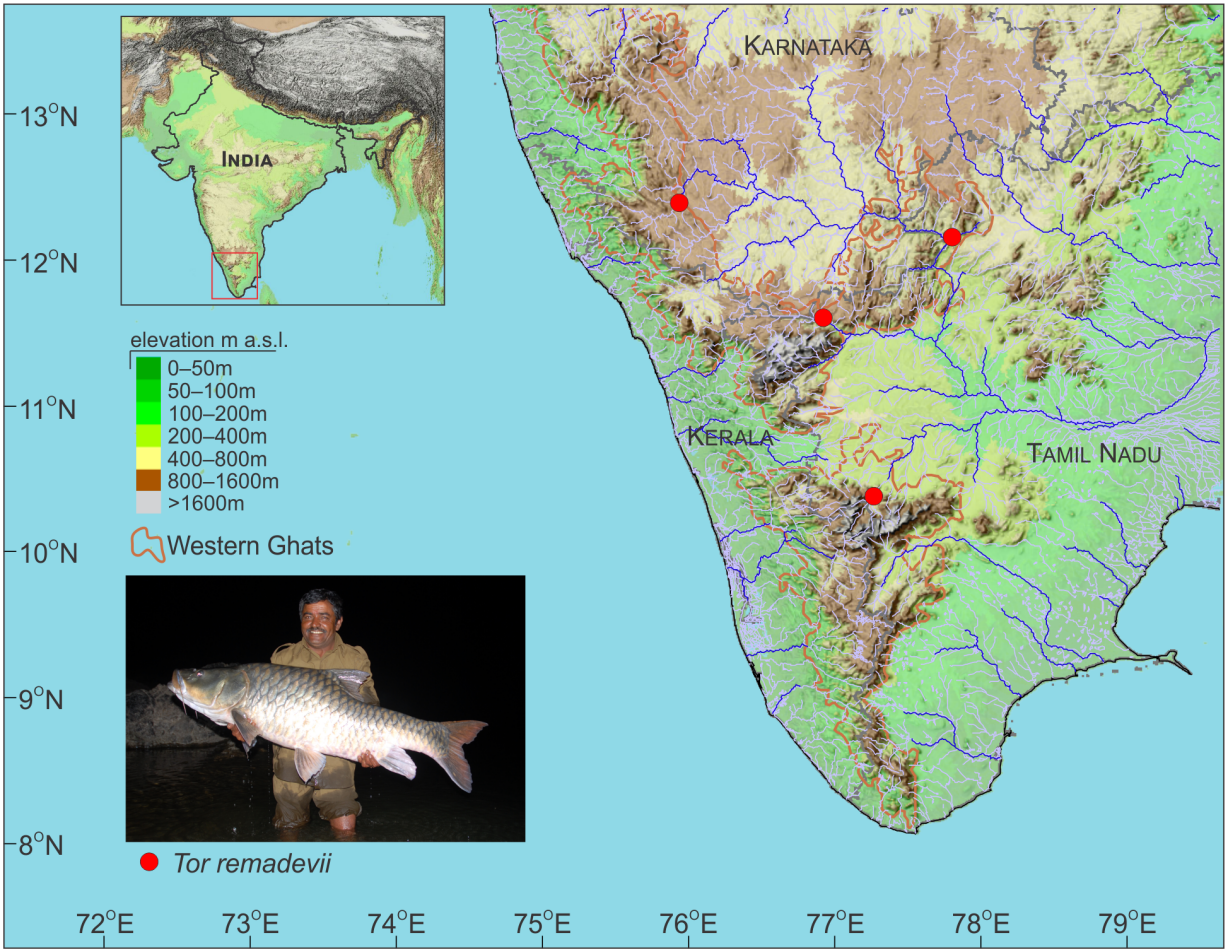
Collection locations of *Tor remadevii* from the tributaries of the River Cauvery, India.

*Tor remadevii* is currently known only from the eastward flowing River Cauvery and its tributaries including the Moyar, Kabini, Bhavani and the Pambar, in the Western Ghats Hotspot of peninsular India (Fig 7).

### Habitat

**Fig 8:**
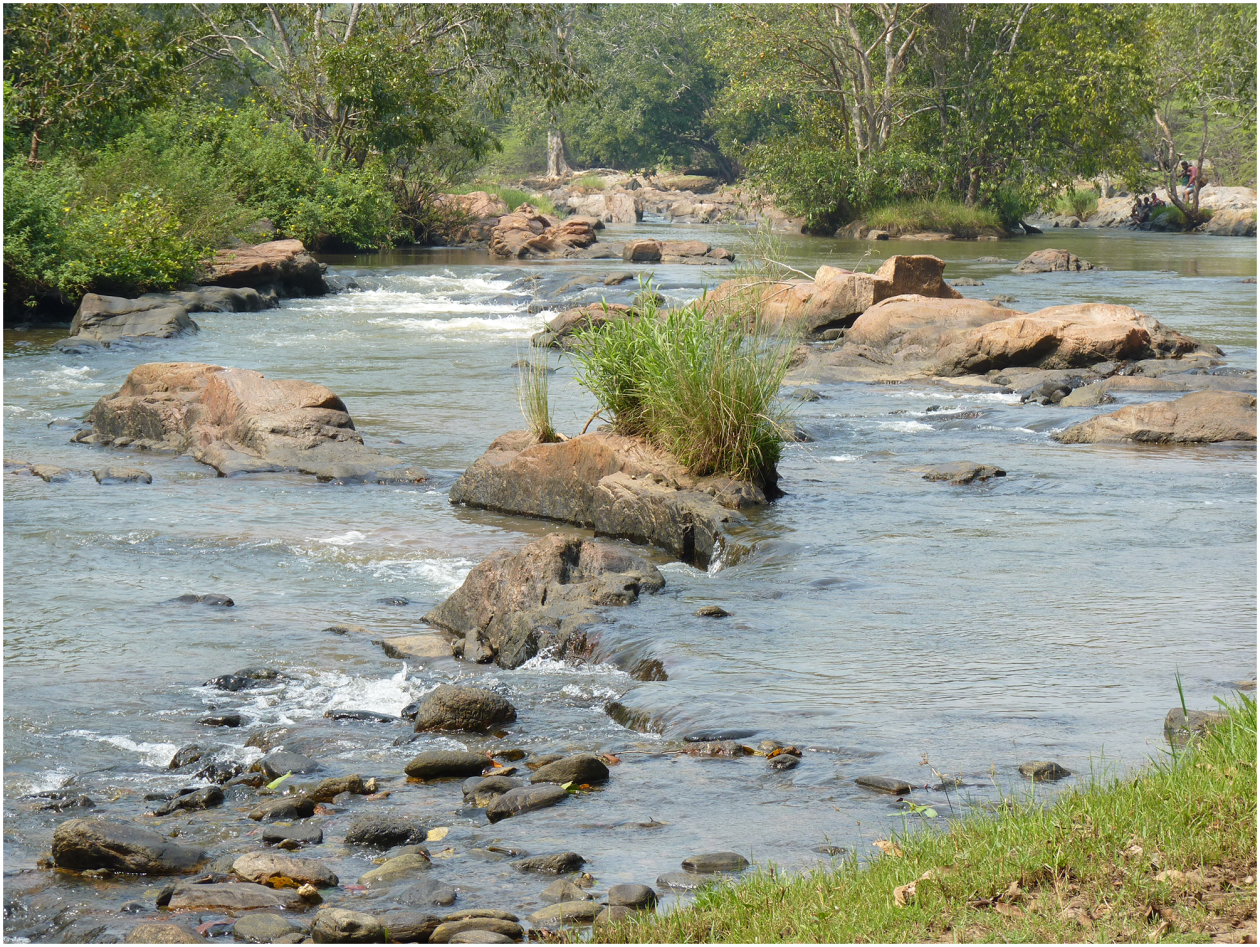
Typical habitat of *Tor remadevii* in the River Moyar, India.

While functional habitats are yet to be elucidated, *T*. *remadevii* inhabits the middle to upper reaches of the River Cauvery and some of its tributaries. Mesohabitat utilization is known to incorporate shallow high velocity rapids to deep, slow flowing pools, with substrates typically composed of bedrock and boulders (Fig 8).

## Discussion

These results confirm that the hump-backed mahseer, an iconic species that can be classed as mega-fauna on account of its large body size, is genetically distinct from other South Indian *Tor* fishes and is conspecific with *T*. *remadevii*. In addition to their potentially large adult body sizes, they can be distinguished from other *Tor* fishes by definitive morphological characters including their inter-orbital distances, distinctive kink in the pre-opercle, a well-developed mandible and orange colouration of the caudal fin. These results also reveal that *T*. *remadevii* only occurs in the River Cauvery basin, and thus appears to be endemic with a limited distribution. Given the on-going threats to their populations in the Cauvery [5], these results highlight that despite their iconic status, *T*. *remadevii* is imperiled and urgent conservation assessments and actions are needed forthwith.

The first documented record of the hump-backed mahseer in scientific literature dates back to 1849, when British naturalist Thomas Jerdon [35] mentioned collecting from Seringapatanam (= Srirangapatanam) in the River Cauvery, a juvenile specimen of a mahseer that grows to enormous sizes, which he identified as *Barbus megalepis*. Later, in a classical work on angling in India [6], Henry Sullivan Thomas characterized this fish as having a deeper body and higher back and called it the Bawwany mahseer, or ‘Barbus tor’. Subsequent workers [10–13] considered Jerdon’s and Thomas’ fish to be synonymous with *Barbus mussullah* Sykes, and called it the hump-backed mahseer [36].

The identity and generic placement of *Barbus mussullah* Sykes, which was long unclear, having been considered a synonym of *Cyprinus curmuca* Hamilton, or a species of *Tor* Gray, was clarified to be a species of *Hypselobarbus* Bleeker and the identity stabilized by the designation of a neotype [15–16]. However, Knight et al. [15–16] also brought attention to the fact that the identity of *Barbus* (*Tor*) *mussullah* sensu Hora [10–11] still remained to be elucidated. Hora’s use of coloration and local knowledge (including local names) to characterize this species [10] was unreliable, as fishes often have a greater variety of local names than any other group of animals [37], with the same name being used for different species and different names being used for the same species. Although there was uncertainty in the use of vernacular names, Hora [10] distinguished the high-backed species, which he called *T*. *mussullah*, from *T*. *khudree* sensu Sykes.

In their work, Knight et al. [15–16] also drew attention to a *Tor* specimen in the unregistered, reserve collections in the Zoological Survey of India, Southern Regional Center, Chennai (ZSI-SRS), labeled *Tor neilli* and originating from the River Krishna at Satara, Maharashtra with a characteristic high back and 24 scales in the lateral series. Knight et al. [15] speculated that this could be the species which Hora [10] considered as *T*. *mussullah*. Quoting Day’s description of *T*. *neilli* from the River Tungabhadra at Kurnool [38], part of the Krishna River basin (from where Hora [10] collected his *T*. *mussullah*), as a large species of mahseer with tubercles on its snout. His illustration of quite a deep-bodied fish, and opinion that this species sometimes has reddish fins, Knight et al [15] suggested that in the event of *T*. *mussullah* sensu Hora [10–11] is found to be a valid, the name *T*. *neilli* should be considered for it.

Comparison of topotypic specimens and/or type material of valid mahseer species of peninsular India (*T*. *malabaricus*, *T*. *khudree* and *T*. *remadevii*) with specimens of the hump-backed mahseer collected from River Cauvery and its tributaries revealed striking similarities between the hump-backed mahseer and *T*. *remadevii* in morphometrics, meristics and mitochondrial DNA (cox1). The *Tor* specimens from the Tungabhadra, a tributary of the Krishna matched topotypic *T*. *khudree* and not the specimens collected in the various tributaries of the Cauvery in their genetic make-up. *Tor neilli* is therefore treated as a junior synonym of *T*. *khudree*, while *T*. *remadevii* is considered as a valid species restricted to the Cauvery River system including its northern and southern tributaries. The name ‘Tor moyarensis’ propagated in popular literature is a ‘nomen nudum’ [39].

The first mention of the name *Tor remadevii* was made in 2007, when Kurup & Radhakrishnan’s description was published in the proceedings of a global mahseer symposium held in Malaysia [17]. Perhaps, because of the limited circulation of this publication, the description went unnoticed, and the same authors published a second paper in the year 2011 [40] reproducing the bulk of the original text, probably with a view to make a ‘formal description’ in a peer reviewed journal. However, the description made in 2007, satisfies all the ‘criteria of availability’ as per the International Code on Zoological Nomenclature (ICZN) (Articles 10, 11, 13 and 16), and therefore the paper published in 2011 [40] is merely a re-description and irrelevant to nomenclature. The original year of publication is 2007, from when the name *T*. *remadevii* became available.

The Catalog of Fishes [41] mentions that the species epithet should be ‘remadeviae’ and not ‘remadevii’ because of the reason that the species was named for K. Rema Devi, (a feminine name). However, the ICZN in its Article 31.2.3 states “If a species-group name (or, in the case of a compound species-group name, its final component word) is not a Latin or latinized word [Articles 11.2, 26], it is to be treated as indeclinable for the purposes of this Article, and need not agree in gender with the generic name with which it is combined (the original spelling is to be retained, with ending unchanged; also see Article 34.2.1)”. Therefore, the correct usage should be *Tor remadevii*.

Having been first brought to the attention of the scientific community in the year 1849 [34], and the recreational angling community in the year 1873 [6], a century and half has since passed before the iconic hump-backed mahseer is afforded a scientific name. With the name now assigned to *T*. *remadevii* and the previously reported imperiled status of this mega-fauna [5], there is an immediate urgency to assess its extinction risk based on the IUCN Red List Categories and Criteria, with a view to affording this iconic species appropriate protection and accelerating the conservation agenda to secure the future sustainability of remaining populations from severe and escalating anthropogenic threats [8].

## Supporting information

S1 Table List of specimens used for the molecular analysis in Fig 3. (DOCX)
