# Supplementary material for: Resolving the taxonomic enigma of the iconic game fish, the hump-backed mahseer from the Western Ghats biodiversity hotspot, India
Source: PLoS One. 2018 Jun 20;13(6):e0199328. doi: 10.1371/journal.pone.0199328 (PMC6010267; doi:10.1371/journal.pone.0199328)
Supplement: S1 Table — (DOCX) [file pone.0199328.s001.docx]

**Table S1. List of specimens used for the molecular analysis in Fig 3**

| Species | Location | GenBank Accession Number |
| --- | --- | --- |
| *Tor remadevii* | Bhavani R | JX401300 |
| *Tor remadevii* | Hognekkal, Cauvery R | KC559903 |
| *Tor remadevii* | Hognekkal, Cauvery R | KC559904 |
| *Tor remadevii* | Hognekkal, Cauvery R | KC559905 |
| *Tor remadevii* | Pambar R | MF591715* |
| *Tor remadevii* | Moyar R | MG769028* |
| *Tor remadevii* | Moyar R | MG769029* |
| *Tor remadevii* | Moyar R | MG769030* |
| *Tor remadevii* | Dubare, Cauvery R | MG769031* |
| *Tor remadevii* | Moyar R | MG769032* |
| *Tor remadevii* | Moyar R | MG769033* |
| *Tor remadevii* | Dubare, Cauvery R | MG769034* |
| *Tor remadevii* | Dubare, Coorg, Cauvery R | MG769035* |
| *Tor remadevii* | Dubare, Coorg, Cauvery R | MG769036* |
| *Tor remadevii* | Dubare, Coorg, Cauvery R | MG769037* |
| *Tor remadevii* | Moyar R | MG769038* |
| *Tor remadevii* | Moyar R | MG769039* |
| *Tor remadevii* | Moyar R | MG769040* |
| *Tor remadevii* | Dubare, Cauvery R | MG769041* |
| *Tor remadevii* | Unknown Location | KM191354 |
| *Tor malabaricus* | Chaliyar R | MG769042* |
| *Tor malabaricus* | Chaliyar R | JX401293 |
| *Tor khudree* | Tungabhadra R | MG769043* |
| *Tor khudree* | Tungabhadra R | MG769044* |
| *Tor khudree* | Indrayani River | MG769045* |
| *Tor khudree* | Krishna R | KJ702318 |
| *Tor khudree* | Krishna R | KP965407 |
| *Tor putitora* | Teesta R | MG769046* |
| *Tor putitora* | Teesta R | MG769047* |
| *Tor putitora* | Teesta R | MG769048* |
| *Tor putitora* | Mahanadi R | HQ609722 |
| *Tor putitora* | Mahanadi R | HQ609723 |
| *Tor* sp. 2 | Harkul Dam | MG769049* |
| *Tor* sp. 2 | Harkul Dam | MG769050* |
| *Tor* sp. 2 | Harkul Dam | MG769051* |
| *Tor* sp. 2 | Vaitarna R | MG769052* |
| *Tor* sp. 2 | Krishna R | MG769053* |
| *Tor* sp. 2 | Krishna R | MG769054* |
| *Tor* sp. 2 | Krishna R | MG769055* |
| *Tor* sp. 2 | Forbes Sagar Lake | MG769056* |
| *Tor* sp. 2 | Gangapur Reservoir | JX260986 |
| *Tor* sp. 2 | Tawa Reservoir | EU714120 |
| *Tor* sp. 1 | Brahmaputra R | KX234717 |
| *Tor* sp. 1 | Ganga R | EU714109 |
| *Tor* sp. 1 | Ganga R | JN965197 |
| *Neolissochilus hexagonolepis* | Chindwin R | KJ909386 |
| *Neolissochilus hexastichus* | Brahmaputra R | JX127239 |
| *Neolissochilus hexastichus* | Brahmaputra R | KJ702137 |

* sequences generated in current study
